# Evaluation of the accuracy, consistency, and scientific reliability of AI-powered Chatbots in endodontic practice

**DOI:** 10.2340/aos.v85.46148

**Published:** 2026-06-04

**Authors:** Saba Kilimci, Elif Delve Başer Can, Jale Tanalp

**Affiliations:** Department of Endodontics, Faculty of Dentistry, Yeditepe University, Istanbul, Turkey

**Keywords:** Artificial intelligence, Chatbots, endodontics, clinical decision-making, guideline adherence

## Abstract

**Objective:**

This study aimed to evaluate the accuracy, consistency, and scientific reliability of two AI-powered chatbots—ChatGPT-3.5 and ChatGPT-4o—in clinical endodontic decision-making, using the recently published *European Society of Endodontology (ESE) S3-level Clinical Practice Guideline* as the gold standard reference.

**Material and Methods:**

Twenty-five dichotomous (yes/no) questions were developed based on the ESE guideline and presented to both chatbots across three time intervals, yielding 300 total responses. Each response was evaluated for accuracy and consistency, and the quality of the supporting references was assessed according to their journal ranking (Q1, Q2, others).

**Results:**

Both ChatGPT versions demonstrated high internal consistency across repeated measurements. ChatGPT-3.5 showed 94.4% agreement (κ = 0.824; 95% confidence interval [CI]: 0.786–0.898; *p* < 0.001), whereas ChatGPT-4o demonstrated 98.9% agreement (κ = 0.937; 95% CI: 0.893–0.965; *p* < 0.001). The accuracy of ChatGPT-3.5 relative to the guideline-based answers was 81.4%, 88.9%, and 82.2% in the morning, afternoon, and evening sessions, respectively, while ChatGPT-4o achieved 82.9%, 83.3%, and 85.4%, respectively. No statistically significant differences were observed between the models across the three time intervals (*p* > 0.05). The proportion of Q1/Q2-ranked references was high and comparable between ChatGPT-3.5 (74–82%) and ChatGPT-4o (76–84%).

**Conclusion:**

Both ChatGPT-3.5 and ChatGPT-4o demonstrated substantial alignment with the ESE S3-level clinical practice guideline. However, these findings should not be interpreted as definitive assessments of current clinical conversational AI systems, and further evaluation of evolving models is required.

## Introduction

“Every aspect of learning or any other feature of intelligence can, in principle, be so precisely described that a machine can be made to simulate it.” [1]. Once a fantasy, artificial intelligence (AI) has become a tangible reality that profoundly influences today’s world. The primary objective of AI is to develop technologies that enable computers to perform tasks intelligently [2]. It relies on algorithms that allow machines to analyze extensive datasets, learn from them, solve problems, adapt, and improve their performance over time [2].

AI has rapidly expanded into dental diagnostics and decision-support systems, also impacting endodontics by broadening its research scope to include diverse applications such as predicting stem cell viability, determining root canal morphology, and detecting root fractures [3]. As dentists have become increasingly reliant on computer programs for clinical decision-making [4–6], such programs must align with current scientific evidence. AI-powered chatbots are subsets of AI systems designed for natural language understanding and generation [7]. A search of the literature reveals that there are approximately 80 studies pertaining to Endodontics, performed using Chat GPT, whose first version was launched in November 2022.

In 2023, the European Society of Endodontology (ESE) released “*Treatment of Pulpal and Apical Disease: The European Society of Endodontology (ESE) S3-level Clinical Practice Guideline,*” providing a robust, evidence-based framework for clinical decision-making [8]. This S3-level guideline represents the highest degree of evidence synthesis currently available in endodontics. Evaluating AI-generated output against this benchmark enables an objective assessment of its reliability as a clinical decision-support tool for practitioners and educators. To date, no study has investigated whether AI-powered chatbots can align with the clinical recommendations of the S3-level guideline.

In this context, given concerns regarding the reliability and consistency of AI systems, the present study aimed to evaluate the accuracy, consistency, and scientific reliability of responses generated by two AI-powered chatbots—ChatGPT-3.5 and ChatGPT-4o—based on the latest ESE guideline [8].

The null hypotheses tested were:

1. There is no statistically significant difference between ChatGPT-3.5 and ChatGPT-4o in terms of guideline-based diagnostic accuracy.
2. ChatGPT responses remain consistent across different times of day.

## Materials and methods

### Ethical approval

Ethical approval was not required for this study, as it did not involve human participants or use any patient data.

### Question design

Two different versions of Chat GPT were included in the present study (ChatGPT-3.5 and ChatGPT-4o). Two expert endodontists developed 25 dichotomous (yes/no) questions, based on the ESE guideline, which were input to both chatbot models [8]. An example question was, “*Is full pulpotomy recommended for patients diagnosed with nontraumatic pulpitis associated with spontaneous pain in permanent teeth?*”.

To ensure methodological transparency and reproducibility, all prompts were standardized as closed-ended clinical questions directly derived from the guideline statements and were presented using identical wording across both chatbot models, without modification. No additional contextual information or prompt engineering techniques were applied.

After receiving the answer from each chatbot, a follow-up question was asked: “*Can you support your answer with scientific evidence?*” This approach enabled the assessment of the presence or absence of supporting evidence and the quality of references provided by each chatbot. As the study aimed to evaluate guideline concordance rather than population parameters, all 25 items from the guideline were included; therefore, a formal sample size calculation was not applicable.

### Generating answers in ChatGPT

The questions were submitted to each chatbot from two separate user accounts, three times per day (morning, afternoon, and evening), using the “new conversation” option each time to avoid contextual bias. A total of 300 individual responses were collected and analyzed.

### Guideline answers and reference categories

The chatbot responses were coded by the researchers as “correct,” “incorrect,” or “unclear,” corresponding to “fully consistent with the guideline recommendation”; “inconsistent or contradicting the guideline”; and “ambiguous, incomplete, or internally inconsistent,” respectively. Responses that were unclear, incomplete, or did not directly address the question were excluded from the analysis to ensure interpretability and methodological rigor. Agreement between the two evaluators was quantified using Cohen’s kappa coefficient. A kappa value of 0.96 was obtained, indicating almost perfect agreement.

To ensure standardization, all prompts were structured as closed-ended clinical scenarios directly derived from guideline statements, with identical wording for both chatbots. References cited by the chatbots were categorized into three groups (Q1, Q2, or others), according to the *Journal Citation Reports* (JCR) classification. All the references provided by the chatbots were checked and confirmed in terms of their existence. All answers were stored in an Excel spreadsheet (Microsoft Corp., Redmond, WA, USA).

### Statistical analysis

Statistical analyses were performed using the NCSS 2007 Statistical Software program (Number Cruncher Statistical System, UT, USA). Alongside using descriptive statistics (mean, standard deviation, median, and interquartile range), the distribution of variables was examined using the Shapiro–Wilk normality test.

The agreement between the two chatbots was evaluated using weighted kappa coefficients, as the same questions were submitted to both models. The consistency of responses obtained at different time intervals (morning, afternoon, and evening) was assessed using Fleiss’ kappa. A gold standard was defined according to the guideline’s dichotomous (yes/no) answers, and the concordance of chatbot responses with this standard was calculated using Weighted kappa.

Differences in accuracy percentages were analyzed using the chi-squared test. The proportion of Q1- and Q2-ranked references among all cited sources was evaluated using the intraclass correlation coefficient (ICC) with 95% confidence intervals. The Q1/Q2 selection percentages between the first and second measurements for each chatbot were compared using the Wilcoxon signed-rank test, while intergroup comparisons between the chatbots were conducted using the Mann–Whitney *U* test.

A sensitivity analysis was conducted by including responses initially classified as unclear or non-interpretable to assess the robustness of the findings and the impact of response interpretability on model performance.

As the study included all available guideline-derived items rather than a sampled population, a formal a priori sample size calculation was not applicable. However, a post hoc power analysis based on the primary between-model accuracy comparison (chi-squared test, α = 0.05, total analyzable responses = 276) showed that the study had 99.3% power to detect a moderate effect size (Cohen’s w = 0.30). *P* < 0.05 was considered statistically significant.

## Results

When comparing the first and second measurements within each chatbot, ChatGPT-3.5 showed 94.4% consistency (κ = 0.824; 95% confidence interval [CI]: 0.786–0.898; *P* < 0.001), whereas ChatGPT-4o demonstrated 98.9% consistency (κ = 0.937; 95% CI: 0.893–0.965; *P* < 0.001), indicating a high level of repeatability for both models (Table 1).

**Table 1:** Consistency and inter-measurement agreement of chatbot responses across different time intervals.

		Value (%)	95% CI (Wald binomial)
**ChatGPT-3.5 Morning/Afternoon/Evening**	**Consistency**	0.944	0.905–0.912
	**Fleiss’ kappa (*p* = 0.0001)**	0.824	0.786–0.898
**ChatGPT-4o Morning/Afternoon/Evening**	**Consistency**	0.989	0.948–0.981
	**Fleiss’ kappa (*p* = 0.0001)**	0.937	0.893–0.965

CI: confidence interval.

Agreement with the guideline-based gold standard ranged between κ = 0.60 and 0.76 across different time intervals, indicating moderate to substantial agreement. Overall, both chatbot models demonstrated consistent alignment with guideline-based recommendations, with no notable differences across time periods (Table 2). In terms of accuracy, ChatGPT-3.5 achieved 81.4%, 88.9%, and 82.2% correct responses in the morning, afternoon, and evening sessions, respectively, while ChatGPT-4o achieved 83.0%, 83.3%, and 85.4%, respectively. No statistically significant differences were observed between the models across time intervals (*p* > 0.05; Table 2). Accuracy estimates were interpreted alongside their corresponding 95% confidence intervals. Consistency across time intervals was further supported by supplementary weighted kappa analyses, which demonstrated high levels of agreement (κW = 0.72–1.00; *p* < 0.001; Table 2).

**Table 2:** Accuracy and agreement of chatbot responses across different time intervals.

Time of day	ChatGPT-3.5 accuracy *n* (%)	ChatGPT-4o accuracy *n* (%)	*P* *	Model agreement (κW)	Gold standard agreement (κ)
**Morning**	35 (81.4)	39 (82.9)	0.844	0.792	0.603
**Afternoon**	40 (88.9)	40 (83.3)	0.636	1.000	0.762
**Evening**	37 (82.2)	41 (85.4)	0.891	0.723	0.613

Accuracy is defined as the proportion of responses consistent with the guideline-based gold standard.

κW: weighted kappa coefficient (model agreement); κ: Cohen’s kappa coefficient (agreement with gold standard).

* Chi-squared test.

A sensitivity analysis was conducted by including responses initially categorized as “unclear” or “non-interpretable.” Of the 300 total responses generated, 24 (8%) were classified as unclear. Including these responses decreased overall accuracy to 74.7% for ChatGPT-3.5 and 80.0% for ChatGPT-4o. However, the comparative performance pattern between the models remained unchanged, and no statistically significant differences were observed (*p* > 0.05), indicating that the findings were robust to the inclusion of ambiguous responses (Table 3).

**Table 3:** Sensitivity analysis of overall accuracy, including unclear responses.*

Total responses (*n*)	Unclear responses (*n*, %)	Accuracy (excluding unclear) (%)	Accuracy (including unclear) (%)
300	24 (8%)	83.3	77.3

* Sensitivity analysis including responses initially categorized as “unclear” or “non-interpretable.” A total of 24 out of 300 responses (8.0%) were classified as unclear. Inclusion of unclear responses resulted in lower overall accuracy, highlighting the potential overestimation of performance when such responses are excluded. No statistical comparison was performed, as this analysis was intended to descriptively assess the impact of including unclear responses.

Direct comparison between the models revealed good agreement in Q1/Q2 reference selection, with ICC values of 0.747 (95% CI: 0.698–0.867), 0.763 (95% CI: 0.716–0.852), and 0.772 (95% CI: 0.711–0.859) across the morning, afternoon, and evening sessions. No statistically significant differences were observed between the models or across time intervals (*p* > 0.05; Table 4).

**Table 4:** Comparison of Q1 and Q2 reference selection rates and agreement between ChatGPT3.5 and ChatGPT-4o across different time intervals.

Time of day	Statistic	ChatGPT-3.5	ChatGPT-4o	ICC (95% CI)	*p* *
Morning	Mean ± SD	82.33 ± 22.41	76.51 ± 22.13	0.747 (0.698–0.867)	0.167
	Median (IQR)	88.89 (75–100)	75 (66.67–100)		
Afternoon	Mean ± SD	74.08 ± 23.27	83.63 ± 20.87	0.763 (0.716–0.852)	0.065
	Median (IQR)	75 (57.5–100)	100 (66.67–100)		
Evening	Mean ± SD	81.61 ± 17.04	80.08 ± 25.2	0.772 (0.711–0.859)	0.796
	Median (IQR)	80 (66.67–100)	100 (58.33–100)		

CI: confidence interval; ICC: intraclass correlation coefficient; IQR: interquartile range; SD: standard deviation.

* Mann–Whitney U test.

## Discussion

This study primarily aimed to determine the compatibility of AI-powered chatbots with the recently published ESE guideline and to evaluate their accuracy, consistency, and reliability in clinical decision-making [8].

ChatGPT-3.5, developed by OpenAI Inc. (San Francisco, CA, USA), is based on a transformer-based model known as the *Generative Pretrained Transformer* (GPT) and trained on diverse internet text using unsupervised learning techniques to enhance contextual understanding and response generation [9]. The model reached 100 million active monthly users in January 2023, just 2 months after it was launched [10]. ChatGPT operates through algorithms that interpret natural language and generate either predefined or dynamically created responses [11]. Its applications span a wide range, from creative writing and programming to scientific content generation and text editing [12, 13].

ChatGPT-4 was launched in March 2023, followed by ChatGPT-4o in May 2024. According to the developer, the updated model provides enhanced multimodal capabilities, enabling the processing and generation of text, audio, and image-based inputs. Furthermore, ChatGPT-4o demonstrates increased processing speed, reduced latency, and improved linguistic accuracy, particularly in non-English languages. According to OpenAI’s official documentation, GPT-4 has a reported knowledge cutoff date of December 1, 2023, while GPT-4o has a reported knowledge cutoff date of October 1, 2023 [14]. At the time this study was conducted, the two different versions used (Chat GPT 3.5 and Chat GPT 4o) were the most recent and available versions, and therefore, they were included in the investigation. The evolution of Chatbots is definitely a very dynamic process, and further studies are warranted to evaluate the capacities of newer versions which display a very speedy development process.

In the present study, 25 questions were derived based on the recommendations outlined in the ESE guideline. The expected yes/no answers were determined by two expert endodontists who reached consensus based on the information provided in the guideline. It was noteworthy that both chatbots produced a similar percentage of correct responses, with ChatGPT-3.5 achieving an average accuracy of approximately 84% and ChatGPT-4o approximately 84%. No significant differences in the percentage of correct answers were generated by chatbots at different times of the day. Furthermore, timing was defined according to local time (GMT +3) solely to assess response repeatability and consistency. As AI chatbots operate on global infrastructures, local time does not necessarily reflect server load or usage patterns, and no such inference was intended.

A unique aspect of the present study was the inclusion of a secondary question following each “yes” or “no” response, prompting the chatbot to provide supporting scientific evidence. When these answers were analyzed, both chatbots yielded statistically similar results, frequently citing references from Q1 and Q2 journals demonstrating the feasibility and accessibility of both models as a valuable tool for evidence-based decision support.

To the authors’ knowledge, this is the first study to evaluate the reliability of contemporary AI chatbots in terms of their compatibility with the recently released ESE guideline. However, previous studies have explored chatbot reliability in general endodontics and other dental disciplines [15–18].

In a related study, Öztürk et al. reported that ChatGPT-4o achieved a significantly higher accuracy rate (92.8%) than ChatGPT-4 [19]. In contrast, the present study found no significant difference between ChatGPT-3.5 and ChatGPT-4o. This discrepancy may be attributed to differences in question type and content. While Öztürk et al. included questions based on well-established textbook knowledge with definitive answers, the present study used questions whose levels of evidence may vary according to emerging literature. Öztürk et al. also emphasized that differences in question types, fields of study, languages, and data source reliability, as well as definitions and calculation methods used for accuracy and consistency, can significantly influence the reported results [19].

Similarly, Ahmad et al. reported superior accuracy for ChatGPT-4o (92.7%) in their study comparing three chatbots in periodontology [20]. Although the discipline examined in their study differed from endodontics, the findings align with the present results regarding the accuracy performance of ChatGPT-4o.

In a study by Krishna et al., ChatGPT-3.5 and ChatGPT-4 demonstrated reliable accuracy across three consecutive attempts. However, both chatbots exhibited limited repeatability and robustness and tended to display overconfidence in their responses [21]. In contrast, the present study found the repeatability of both chatbots to be acceptable, with no significant differences observed in the percentage of correct answers across different times of the day. This discrepancy may be attributed to differences in the disciplines assessed and to the use of chatbot versions released by the same developer in the current investigation.

Ozden et al. evaluated the consistency and accuracy of responses generated by ChatGPT and Google Bard (Gemini) to questions related to dental trauma derived from the *International Association of Dental Traumatology (IADT) Guidelines* [22]. Both applications provided correct answers to 57.5% of the questions, indicating that the overall reliability and accuracy of these chatbots in addressing dental trauma queries remain limited. The similarity between their study and the present investigation lies in the fact that both sets of questions were derived from internationally recognized clinical guidelines.

The present study has several inherent limitations that should be addressed in future research. The question set could be expanded to include a greater variety of formats, multiple repetitions and extending assessment could be performed to enhance the reliability of the findings. Additionally, a more specific categorization of questions—focusing on individual treatments or clinical procedures—may provide a more precise evaluation of chatbot performance. Furthermore, the primary outcome was based only on interpretable responses, which may have led to an overestimation of real-world clinical usability, as ambiguous or non-actionable outputs are also relevant in clinical practice.

Although a dichotomous (yes/no) question framework was intentionally adopted to ensure standardization, reproducibility, and objective comparison with the guideline-based gold standard, it may not fully capture the complexity of real-world clinical decision-making. Clinical reasoning often involves varying levels of evidence, contextual interpretation, and uncertainty, which cannot be adequately represented by binary outcomes. Consequently, the absence of statistically significant differences between the models should be interpreted with caution, as subtle differences in reasoning depth and clinical nuance may have been masked by the dichotomous design. In addition, the use of expert consensus to define the gold standard may not fully reflect variability in clinical judgment or potential differences in guideline interpretation across practitioners. Future studies incorporating more nuanced or open-ended question formats may provide a more comprehensive evaluation of chatbot clinical reasoning.

A key limitation of this study is that responses classified as “unclear” were excluded from the accuracy and consistency analyses. While this approach was adopted to ensure analytical clarity and avoid subjective interpretation of ambiguous outputs, it may have led to an overestimation of chatbot performance. The reported accuracy rates thus reflect performance conditional on interpretable responses, rather than the overall probability of providing a correct answer to a clinical question. Given that ambiguous, incomplete, or evasive responses are clinically relevant behaviors in generative models, this distinction should be considered when interpreting the findings and their potential clinical implications. The sensitivity analysis, including unclear responses, demonstrated lower overall accuracy while preserving the comparative performance pattern between models, underscoring the impact of response interpretability on performance estimates.

Another important limitation concerns the assessment of reference quality. Although all references generated by the chatbots were manually verified for existence, the evaluation of reference quality was primarily based on JCR quartile classification. This approach does not allow for a detailed assessment of the relevance of the cited article to the specific clinical question, the appropriateness of the study design, or the accuracy of the linkage between the chatbot’s clinical statement and the referenced source. Therefore, the reported findings regarding reference quality should be interpreted as indirect indicators rather than as a comprehensive validation of the scientific soundness of the chatbot outputs. Future studies incorporating more granular content-level analyses would strengthen transparency and reproducibility.

Another limitation of this study is that the clinical guideline used as the reference standard is publicly available online. As large language models may have been trained on publicly accessible data sources, including clinical guidelines, the potential for data contamination cannot be excluded. This may have contributed to an overestimation of chatbot performance, as the models may have been previously exposed to similar or identical content. Therefore, the findings should be interpreted with caution, particularly when considering the apparent alignment between chatbot responses and guideline recommendations.

The absence of domain-specific subgroup analyses represents a limitation of this study. As the questions were not categorized according to specific clinical domains, potential variations in chatbot performance across different areas of endodontic practice could not be evaluated. Future studies incorporating domain-specific analyses may provide a more detailed understanding of chatbot performance across different clinical decision-making contexts.

An additional limitation relates to the inclusion of ChatGPT-3.5, which, although appropriate at the time of study design and data collection, has since been superseded by more recent model iterations. Advances in model architecture, training strategies, and alignment techniques in newer conversational AI systems may result in materially different performance profiles. More broadly, the chatbot versions evaluated in this study reflect the state of large language models at the time of data collection. Given the rapid evolution of AI systems, the findings should be interpreted as model-specific and time-sensitive, rather than as a definitive evaluation of current conversational AI systems in clinical practice.

Emphasis should also be made on the fact that the use of Chatbots in Endodontics as well as dentistry in general is still an evolving topic which is open to improvements and modifications in methodology. As further research is gathered in the literature, we will have a better understanding of the fundamental principles of Chatbot studies and more standardized methodologies may be introduced.

Considering the continuously evolving nature of clinical guidelines, future researches would provide valuable insights into the longitudinal stability and reliable integration of AI models into dental organizations, therefore, representing a significant advancement in the digital era.

## Conclusion

Within the limitations of this study, both ChatGPT-3.5 and ChatGPT-4o demonstrated high levels of accuracy and consistency with the first S3-level clinical practice guideline in endodontics. While the results highlight important strengths and limitations, they should not be interpreted as definitive assessments of current state-of-the-art clinical conversational AI systems. Continued evaluation of evolving models will be essential to determine their real-world clinical reliability and applicability.

## Data Availability

All data generated or analyzed during this study are included in this published article.
